# Targeted optimization of central carbon metabolism for engineering succinate production in *Escherichia coli*

**DOI:** 10.1186/s12896-016-0284-7

**Published:** 2016-06-24

**Authors:** Ying Zhao, Chang-Song Wang, Fei-Fei Li, Zhen-Ning Liu, Guang-Rong Zhao

**Affiliations:** Department of Pharmaceutical Engineering, School of Chemical Engineering and Technology, Tianjin University, Tianjin, 300072 China; Key Laboratory of Systems Bioengineering, Ministry of Education, Tianjin, 300072 China; SynBio Research Platform, Collaborative Innovation Center of Chemical Science and Engineering, Tianjin, 300072 China; Present address: PPG Coating (Tianjin) Co., Ltd. Tianjin Economic Technological Development Area (TEDA), 192 Huanghai Road, Tianjin, 300457 China

**Keywords:** Succinate, *Escherichia coli*, sRNA, Metabolic engineering, Synthetic biology

## Abstract

**Background:**

Succinate is a kind of industrially important C4 platform chemical for synthesis of high value added products. Due to the economical and environmental advantages, considerable efforts on metabolic engineering and synthetic biology have been invested for bio-based production of succinate. Precursor phosphoenolpyruvate (PEP) is consumed for transport and phosphorylation of glucose, and large amounts of byproducts are produced, which are the crucial obstacles preventing the improvement of succinate production. In this study, instead of deleting genes involved in the formation of lactate, acetate and formate, we optimized the central carbon metabolism by targeting at metabolic node PEP to improve succinate production and decrease accumulation of byproducts in engineered *E. coli*.

**Results:**

By deleting *ptsG*, *ppc*, *pykA*, *maeA* and *maeB*, we constructed the initial succinate-producing strain to achieve succinate yield of 0.22 mol/mol glucose, which was 2.1-fold higher than that of the parent strain. Then, by targeting at both reductive TCA arm and PEP carboxylation, we deleted *sdh* and co-overexpressed *pck* and *ecaA,* which led to a significant improvement in succinate yield of 1.13 mol/mol glucose. After fine-tuning of *pykF* expression by anti-*pykF* sRNA, yields of lactate and acetate were decreased by 43.48 and 38.09 %, respectively. The anaerobic stoichiometric model on metabolic network showed that the carbon fraction to succinate of engineered strains was significantly increased at the expense of decreased fluxes to lactate and acetate. In batch fermentation, the optimized strain BKS15 produced succinate with specific productivity of 5.89 mmol gDCW^−1^ h^−1^.

**Conclusions:**

This report successfully optimizes succinate production by targeting at PEP of the central carbon metabolism. Co-overexpressing *pck*-*ecaA,* deleting *sdh* and finely tuning *pykF* expression are efficient strategies for improving succinate production and minimizing accumulation of lactate and acetate in metabolically engineered *E. coli*.

**Electronic supplementary material:**

The online version of this article (doi:10.1186/s12896-016-0284-7) contains supplementary material, which is available to authorized users.

## Background

Succinate, an important member of C4-dicarboxylic acid family, has been widely used in agricultural, food, pharmaceutical, cosmetic, textile and fine chemicals industries [1, 2]. Meanwhile, succinate has received considerable attention to synthesize various valuable molecules such as 1,4-butanediol, tetrahydrofuran, γ-butyrolactone and adipic acid [3]. Petrochemistry-based succinate production requires various metal catalysts and discharges organic wastes, which make petrochemical processes costly and not environmental friendly. Bio-based succinate production is a promising and green process as it uses renewable bioresources as substrates and fixes greenhouse gas CO_2_ [4]. Therefore, the concomitant economical and environmental advantages stimulate the efforts to engineer microorganisms for efficient succinate production.

Succinate can be naturally produced by many strict anaerobic bacteria and facultative anaerobes. *Escherichia coli* is most widely studied for succinate production due to its convenience for genetic manipulation and fast growth with flexible nutrient requirements [5]. However, the wild *E. coli* strain prefers to produce lactate and acetate as major products with a small amount of succinate in mixed-acid fermentation under anaerobic conditions [6]. Efforts of metabolic engineering and adaptive evolution have been made to obtain succinate-producing *E. coli*. Inactivation of genes accounting for biosyntheses of those byproducts was first pursued to produce succinate as the predominant fermentation product. However, the mutant *E. coli* strains deficient in *ldhA* (coding lactate dehydrogenase) and *pflB* (coding pyruvate-formate lyase), *adhE* (coding alcohol dehydrogenase) and *pta* (coding phosphotransacetylase) or their combinations were unable to anaerobically grow on glucose media and the titer and yield of succinate were relatively low. For example, the mutant *E.coli* strain NZN111 deficient in *ldhA* and *pflB* only produced minor amount of succinate [7]. Evolutionary engineering of strain NZN111 led to spontaneous chromosomal mutant strain AFP111, which was able to ferment glucose anaerobically and produced higher succinate yield, as well as higher acetate [8]. Similarly, by combining metabolic engineering and evolution of over 2000 generations screened on glucose minimal medium, *E. coli* strain KJ073 with deletions of *ldhA*, *adhE*, *ackA* (coding acetate kinase), *focA* (coding formate channel), *pflB*, *mgsA* (coding methylglyoxal synthase) and *poxB* (coding pyruvate oxidase) was capable of producing high succinate yield, but significant amounts of acetate and malate were also produced [9].

Metabolic targets of the central carbon metabolism have been used to improve succinate production in *E. coli*. In order to enhance carbon flux to succinate, formation of oxaloacetate (OAA) from pyruvate or phosphoenolpyruvate (PEP) was chosen as metabolic target. Heterologous expressions of *pyc* (coding pyruvate carboyxlase, PYC) from *Rhizobium etli* [10] or from *Lactococcus lactis* [11, 12], *pck* (coding PEP carboxykinase, PCK) from *Actinobacillus succinogenes* [13, 14] and overexpression of native *ppc* (coding PEP carboxylase, PPC) [15] were shown to increase succinate production in recombinant *E. coli* strains. Subtle co-overexpression of both *ppc* and *pck* genes regulated by promoters with different strengths improved succinate production [16]. To increase NADH availability in succinate-producing *E. coli*, several genes involved in redox reactions were identified to improve cell growth impairment under microaerobic conditions [17]. Heterologous NAD^+^-dependent formate dehydrogenase gene *fdh* of *Candida boidinii* or native nicotinate phosphoribosyltransferase gene *pncB* were co-overexpressed with *Lactococcus lactis pyc* gene to achieve the redox and ATP balance [18, 19]. Activation of pentose phosphate pathway, transhydrogenase and pyruvate dehydrogenase were identified for improved succinate production by increasing reducing power supplement [20]. To enhance glucose utilization in *E. coli* strain deficient in PEP carbohydrate phosphotransferase system (PTS), native *galP* (coding D-galactose transporter) and *glk* (coding glucokinase) were co-overexpressed or modulated to facilitate succinate production [21]. *Zymomonas mobilis glf* gene (coding glucose facilitator, Glf) was more efficient than *E. coli galP* gene due to the higher transport velocity and lower energetic cost of Glf [22]. In addition, C4-dicarboxylic acid transporter genes were also activated to decrease the feedback effects through accelerating succinate export [23, 24].

**Fig. 1:**
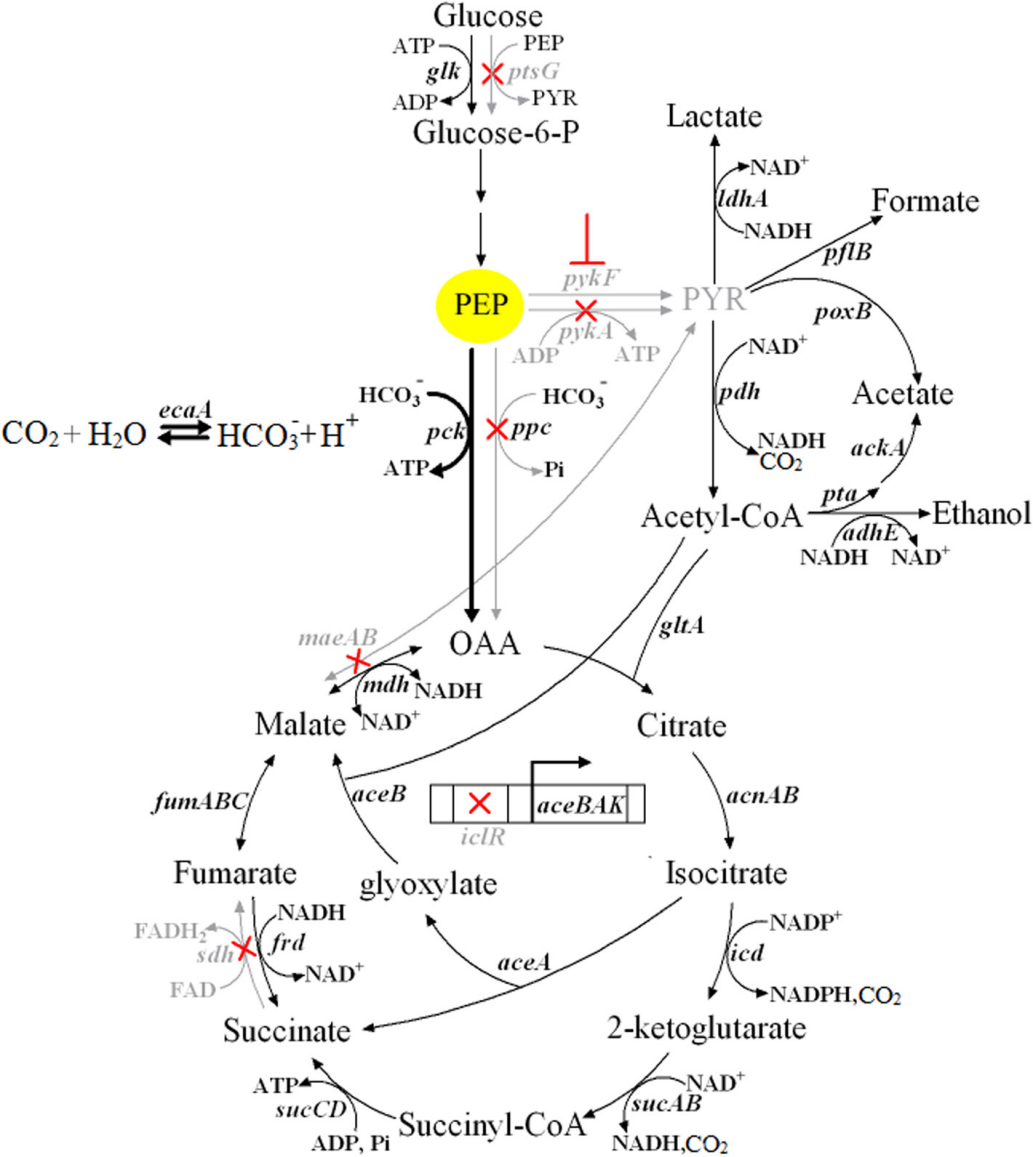
Targeted engineering of the central carbon metabolism for succinate production in *E. coli*. Red crosses represent deletion of gene and the reactions affected by the deletion are indicated with grey. The black arrows of the reactions involved in the overexpressed genes are thickened. Red ┫ represents inhibition of *pykF* expression by anti-*pykF* sRNA either on high-copy-number plasmid (pRSFDuet-1) (H) or low-copy-number plasmid (pBldgbrick2) (L). Genes coding the corresponding enzymes in the pathways: *ptsG*, glucose phosphotransferase; *pykF*, pyruvate kinase I; *pykA*, pyruvate kinase II; *ppc*, PEP carboxylase; *pck*, PEP carboxykinase; *ecaA*, carbonic anhydrase; *iclR*, transcriptional repressor IclR; *aceA*, isocitrate lyase; *aceB*, malate synthase; *aceK*, isocitrate dehydrogenase kinase/phosphatase; *ldhA*, lactate dehydrogenase; *pflB*, pyruvate formate lyase; *pdh*, pyruvate dehydrogenase; *poxB*, pyruvate oxidase; *pta*, phosphotransacetylase; *ackA*, acetate kinase; *adhE*, alcohol/acetaldehyde dehydrogenase; *maeAB*, malic enzyme; *mdh*, malate dehydrogenase; *fumABC*, fumaraseABC; *frd*, fumarate reductase; *sdh*, succinate dehydrogenase; *sucABCD*, succinyl CoA synthase; *icd*, isocitrate dehydrogenase; *acnAB*, aconitate hydratase; *gltA*, citrate synthase

Although considerable metabolic targets are available to improve succinate production, genes involved in competing pathways such as the formation of lactate, acetate, formate and ethanol were inactivated in previous works. In this study, targeted engineering strategy was employed to optimize metabolic pathway of succinate production from glucose without deletions of *ldhA*, *pflB*, *pta*-*ackA*, and *adhE* (Fig. 1). Focusing on PEP node as the engineering target, metabolic flux from PEP was enhanced to OAA and minimized to lactate and acetate. By pentuple deletions of genes *ptsG* (coding glucose phosphotransferase), *pykA* (coding pyruvate kinase II), *ppc*, *maeA* and *maeB* (coding malic enzymes) of the central carbon metabolism, we reconstructed initial *E. coli* strain to increase PEP pool for succinate production. Then we optimized metabolic flux to succinate from PEP by deletion of *sdh* (coding succinate dehydrogenase) and *iclR* (coding transcriptional repressor IclR) as well as co-overexpression of *pck*-*ecaA* (coding carbonic anhydrase). We further attenuated the accumulation of lactate and acetate by fine tuning of *pykF* (coding pyruvate kinase I) expression via antisense sRNA strategy to prevent metabolic flux to pyruvate from PEP. Finally, the fermentation process was carried out with optimized succinate-producing strains.

## Results and discussion

### Initial construction for succinate production

**Fig. 2:**
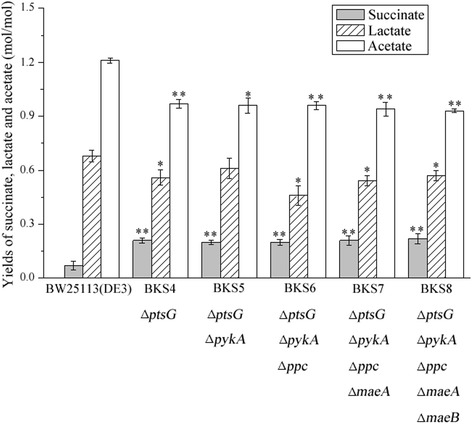
Yields of succinate, lactate and acetate of initial succinate-producing strains. Error bars represent SD for three replicates. Asterisks indicate *p*-values (**p < 0.01, *p < 0.05) compared to BW25113 (DE3)

The wildtype *E. coli* BW25113 (DE3) produced a small amount of succinate in the acid mixture (Fig. 2) from glucose under anaerobic fermentation conditions, which was consistent with the previous report [6]. Glucose uptake through PTS system consumes almost half of the available PEP that is the precursor of succinate, which leads to the significantly decreased amounts of PEP for succinate production. In *E. coli*, the inactivation and mutation of genes involved in the PTS system was beneficial for succinate production [25, 26]. Thus, to save PEP from consumption of PTS system, we deleted *ptsG* gene in strain BW25113 (DE3) and constructed strain BKS4. Succinate production of strain BKS4 was significantly increased with 2.0-fold higher yield than that of strain BW25113 (DE3) (*p* < 0.01) (Fig. 2). Meanwhile, the yields of lactate and acetate in strain BKS4 were decreased by 17.65 % (*p* < 0.05) and 19.83 % (*p* < 0.01), respectively. The results indicated that the inactivation of PTS system played an essential role in the availability of PEP to support succinate production.

In succinate metabolic pathway, the carboxylation of PEP catalyzed by PPC or PCK is a rate-limiting step committed to succinate production. ATP is essentially consumed for PPC catalyzing the formation of OAA from PEP [27]. On the contrary, one molecule ATP is generated from carboxylation of one molecule PEP catalyzed by PCK. The deletion of *pck* gene in *E. coli* remarkably inhibited succinate production as well as the cell growth [27], indicating that PCK might be more efficient than PPC. In addition, the function of PCK was partially inhibited by PPC under anaerobic fermentation [13, 14]. Thus, we deleted *ppc* gene to enhance energy supplement and activate PCK. Furthermore, both PEP and malate would convert to pyruvate, which is smoothly turned into byproducts lactate, acetate and formate via the decarboxylation, dehydrogenation, and pyruvate-formate lyase, respectively. Formate is further split into carbon dioxide and water by formate dehydrogenase, while lactate and acetate accumulate in fermentation broth. Since the substrate specificity of malic enzymes for malate is 6-fold higher than that for pyruvate, malic enzymes encoded by *maeA* and *maeB* tend to catalyze the decarboxylation of malate to pyruvate [28]. The formation of pyruvate and its derivative byproducts strongly compete with succinate production for PEP and malate. Inactivation of *pykA* and *pykF* has been shown to be effective in inhibiting the conversion of PEP to pyruvate [29]. Consequently, in order to inhibit the formation of pyruvate from PEP and malate, we deleted *pykA*, *maeA* and *maeB* genes. Unfortunately, compared to strain BKS4, strain BKS8 with deletion of *pykA*, *ppc*, *maeA* and *maeB* did neither significantly attenuate the accumulation of lactate and acetate, nor increase the succinate yield (Fig. 2). The low expression level of *pck* gene in wild-type *E. coli* could result in the insufficient metabolic flux to OAA [27], and *pykF* might be more active than *pykA* in the formation of pyruvate from PEP. It suggested that *pck* and *pykF* genes could be the potential targets. Therefore, using initial strain BKS8, we further optimize these two targets of succinate metabolic pathway to improve succinate production.

### Combined optimization of targeting at TCA cycle and carboxylation of PEP to increase succinate production

Succinate, an essential intermediate of TCA cycle, cannot be efficiently accumulated in *E. coli* fermentation. In order to increase succinate production, we optimized succinate metabolic pathway by preventing the backflow of succinate to fumarate, activating glyoxylate shunt bypass to decrease the requirement of reducing power, and co-overexpressing *pck*-*ecaA* to fix CO_2_ more efficiently.

**Fig. 3:**
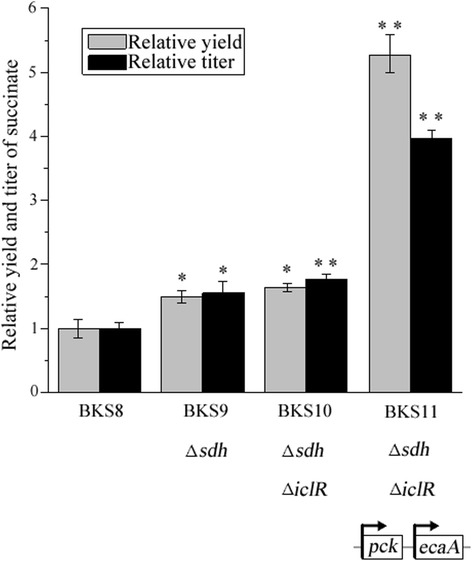
Deletion of *sdh* and *iclR*, and co-overexpression of *pck*-*ecaA* increased succinate production. Error bars represent SD for three replicates. Asterisks indicate *p*-values (**p < 0.01, *p < 0.05) in which BKS9 and BKS10 were compared to BKS8 and BKS11 was compared to BKS10

Succinate dehydrogenase (SHD) encoded by *sdh* gene catalyzes the dehydrogenation of succinate to fumarate. The *sdh* expression was not totally inhibited under anaerobic conditions [30]. Herein, we deleted *sdh* gene to enhance the reductive TCA arm and block the conversion of succinate to fumarate in strain BKS8 background. As expected, the titer and yield of succinate in strain BKS9 were increased by 55.24 % (7.11 mM) (*p* < 0.05) and 50.00 % (0.33 mol/mol glucose) (*p* < 0.05), respectively (Fig. 3). The inactivation of *sdh* gene showed to increase succinate production in *E. coli* and *Corynebacterium glutamicum* under aerobic conditions [31–33]. To the best of our knowledge, *sdh* gene was first deleted to improve anaerobic succinate production in our study.

Glyoxylate shunt bypass could recover the metabolic flux of the oxidative TCA arm and acetyl-CoA of pyruvate metabolism with less reducing power used, and might contribute to succinate production. The *aceBAK* operon coding isocitrate lyase, malate synthase and isocitrate dehydrogenase kinase is responsible for the glyoxylate shunt bypass. The transcription of the *aceBAK* operon is tightly repressed by transcription factor IclR, but induced by inactivating *iclR* gene [34]. Thus, the deletion of *iclR* gene resulted in strain BKS10. As shown in Fig. 3, the titer and yield of succinate in strain BKS10 was not apparently increased. It was likely that the gene expression involved in glyoxylate bypass are complex and regulated by multiple factors [35] and deletion of *iclR* was not sufficient for activating glyoxylate shunt bypass [36]. Conversion of PEP to OAA in succinate metabolic pathway is net carbon integrated via CO_2_ fixation catalyzed by PCK. In fact, the active substrate for PCK is not CO_2_, but the chemically less reactive bicarbonate anion (HCO_3_^−^) [37]. Thus, CaCO_3_, MgCO_3_ or NaHCO_3_ were often added to the culture media. CO_2_ is more permeable across cell membrane than HCO_3_^−^, but the hydration reaction rate of CO_2_ to HCO_3_^−^ is relatively slow. There might not be enough HCO_3_^−^ spontaneously made in vivo to access succinate production. Carbonic anhydrase encoded by *ecaA* gene catalyzes the hydration of intracellular CO_2_ to HCO_3_^−^. Expression of *ecaA* gene of cyanobacterium *Anabaena* in *E. coli* led to an obvious increase in succinate production [38, 39]. Thus, the *ecaA* gene was co-expressed with *pck* in strain BKS10, generating strain BKS11. Compared to strain BKS10, combinatorial expression of *pck*-*ecaA* in strain BKS11 resulted in a 2.2-fold increase in succinate yield (1.16 mol/mol glucose) (*p* < 0.01) and a 1.2-fold increase in succinate titer (18.17 mM) (*p* < 0.01) (Fig. 3).

### Fine tuning of *pykF* expression to improve succinate production

**Fig. 4:**
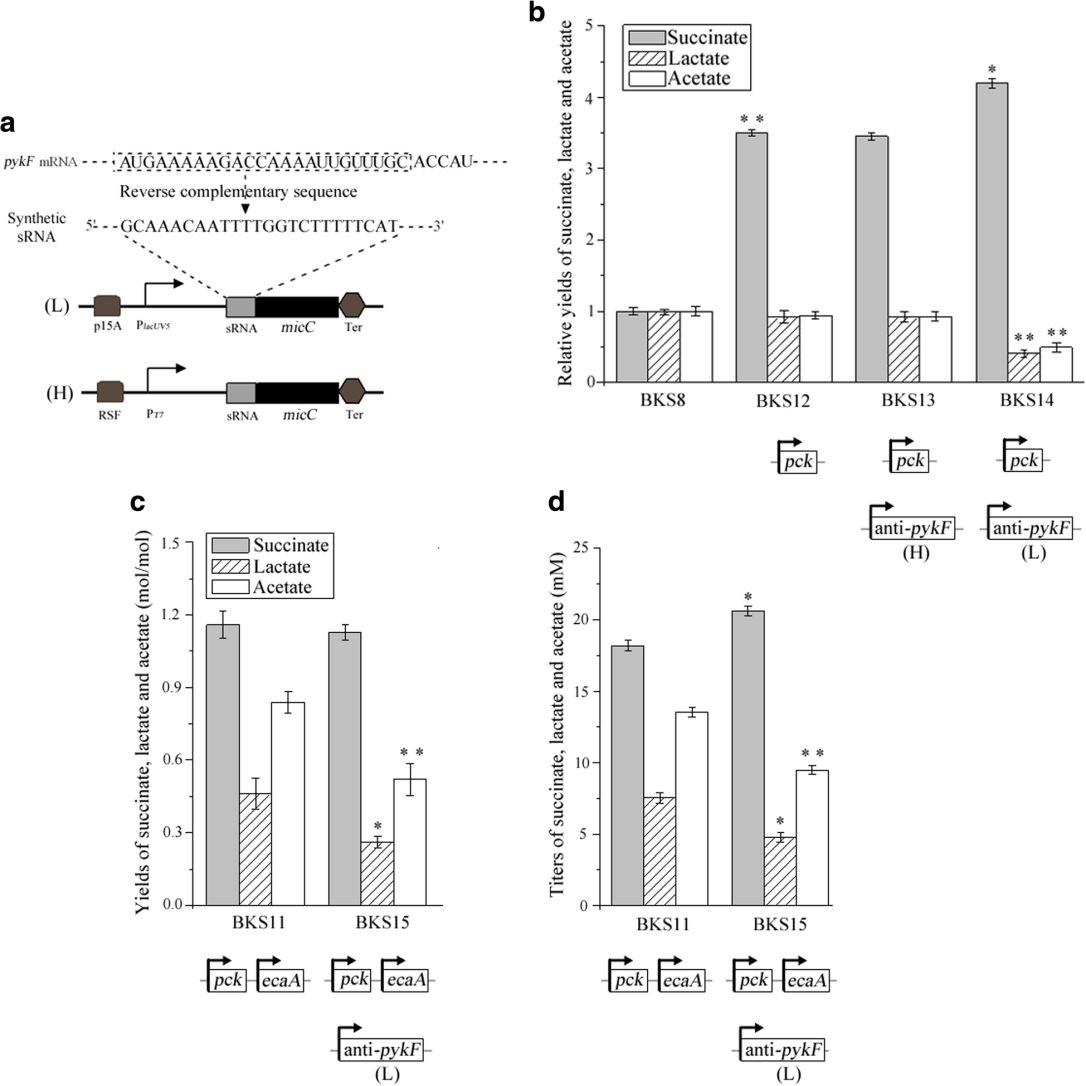
Fine tuning of *pykF* expression strength to improve succinate production and attenuate accumulation of lactate and acetate. **a** Two anti-*pykF* sRNA plasmids were designed and constructed at different expression levels by combinations of promoters and plasmid copy number. (H) and (L) represented high-copy-number plasmid (pRSF) and low-copy-number plasmid (pBldgbrick2), respectively. **b** Relative yields of succinate, lactate and acetate. BKS12 was compared to BKS8 and BKS13 and BKS14 were compared to BKS12. **c** Yields of succinate, lactate and acetate. The significance was compared to BKS11. **d** Titers of succinate, lactate and acetate. The significance was compared to BKS11. Error bars represent SD for three replicates. Asterisks indicate *p*-values (**p < 0.01, *p < 0.05)

Although succinate production was increased remarkably in engineered strains, the yields and titers of lactate and acetate remained high by using the strategies aforementioned in the text (Fig. 4b, c, d), which suggested that metabolic flux from PEP to pyruvate was relatively strong. Deletion of *maeA* and *maeB* and *pykA* did not significantly attenuated the accumulation of lactate and acetate (Fig. 2), suggesting that *pykF* gene might dominate the formation of pyruvate. Thus the strategy of synthetic small RNA (sRNA) engineering [40] was used to finely tune the expression of *pykF* to attenuate the accumulation of lactate and acetate.

Using AUG to nucleotide +24 of the *pykF* mRNA as the binding sequence and selecting *E. coli micC* as the scaffold, anti-*pykF* sRNA working sequence was designed (Fig. 4a). We used two kinds of plasmids with different copy number and tested the inhibitory effects of anti-*pykF* sRNA on the accumulation of lactate and acetate in strain BKS12 with overexpression of *pck* gene. When anti-*pykF* sRNA was expressed on the high-copy-number plasmid pRSF and under the control of T7 promoter, no obvious changes were observed in the yields of succinate, lactate and acetate (Fig. 4b). Then, we constructed the low-copy-number plasmid pBldg-anti-pykF with a pY15A origin of replication, and expression of anti-*pykF* was controlled under *lacUV5* promoter. The metabolite analysis of engineered strain BKS14 showed that the yields of lactate and acetate were decreased by 55.77 % (*p* < 0.01) and 47.73 % (*p* < 0.01), respectively, and the yield of succinate was increased by 23.38 % (*p* < 0.05) compared to BKS12(Fig 4b).

We further tested whether the expression of anti-*pykF* under the control of *lacUV5* promoter in strain BKS11 would improve succinate production and attenuate accumulation of byproducts. pBldg-anti-pykF was transformed into strain BKS11, generating strain BKS15. Compared to strain BKS11, the low expression of anti-*pykF* in strain BKS15 led to the decrease of 43.48 % (*p* < 0.05) and 38.09 % (*p* < 0.01) in the yields of lactate and acetate, respectively (Fig 4c). Although succinate yield of strain BKS15 was not improved, succinate titer was increased by 13.43 % (*p* < 0.05) (Fig. 4d). The results showed that the down-regulated formation of pyruvate by expressing anti-*pykF* would enhance the metabolic flux from PEP to succinate.

### Distribution of intracellular metabolic flux

**Fig. 5:**
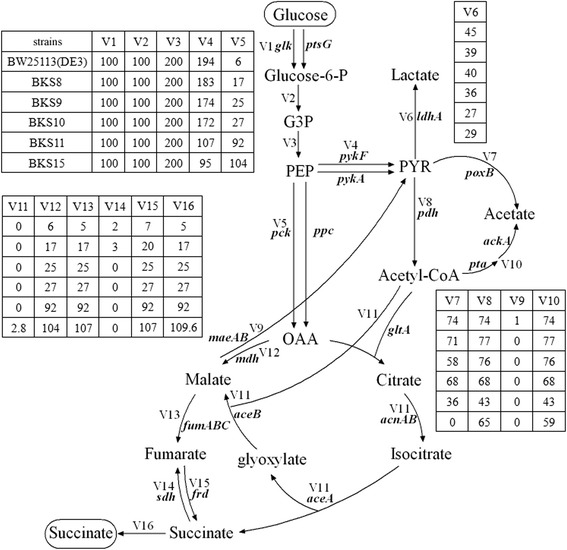
Metabolic flux analysis of succinate-producing strains. The fluxes in mM gDCW^−1^ h^−1^ were calculated according to fermentation data at 40 h and normalized by glucose consumption rate as well as expressed in a basis of 100

Genetic and metabolic modification used in this study remarkably increased succinate production and attenuated the accumulation of lactate and acetate. However, the intracellular metabolic flux distribution of the metabolic network was still unclear. In order to demonstrate in detail how previous efforts changed the metabolic flux directions and optimized the performance of succinate-producing strains step by step, global metabolic flux analysis was made. The simplified metabolic model that described the metabolic relationship in anaerobically fermentative *E. coli* was shown in Fig. 5. This model was comprised of fifteen intermediates and sixteen metabolic reactions designated by V1-V16 (Additional file 1: Table S1). Among these sixteen reactions, the measurable quantities V1, V6, V16 and (V7 + V10) were used to calculate the metabolic fluxes of other intermediates according to relationships shown in Additional file 1: Table S2. The estimated metabolic fluxes in mM gDCW^−1^ h^−1^ of *E. coli* stains BW25113(DE3), BKS8, BKS9, BKS10, BKS11 and BKS15 under anaerobic fermentation were presented in Additional file 1: Table S3.

As shown in Fig. 5, metabolic modifications led to the fact that fluxes to OAA (V5), malate (V12), fumarate (V13), succinate (V15 and V16) were significantly increased and that fluxes to pyruvate (V4), lactate (V6), and acetate (V7 + V10) were remarkably decreased from strains BW25113(DE3) to BKS15. The results indicated that our strategies favored the improvement of succinate production and the decrease of byproduct accumulation.

The split ratios of fluxes to OAA, PYR, lactate, acetate and succinate were obtained by analyzing the PEP, PYR, acetyl-CoA and succinate nodes. As shown in Table 1, compared to strain BW25113 (DE3), the fraction of the metabolic flux diverted to OAA from PEP node (V5/V3) in strain BKS8 increased by 1.8-fold (*p* < 0.01), corresponding 2.2-fold fraction increase of the metabolic flux to succinate (V16/V3) (*p* < 0.01). Pentuple deletions of *ptsG*, *ppc*, *pykA*, *maeA* and *maeB* could significantly streamline PEP pool for succinate production. Strain BKS9 showed the increase of the metabolic flux to succinate (V16/V3), indicating the deletion of *sdh* gene resulted in more metabolic flux to OAA from PEP node (V5/V3). Strain BKS10 did not show carbon flux through glyoxylate shunt bypass (V11 = 0) in the stoichiometric model, indicating that deletion of *iclR* gene did not activate glyoxylate shunt bypass.

**Table 1:** Split ratios of fluxes to OAA, PYR, lactate, acetate and succinate

Strains	Fraction of PEP to OAA (V5/V3)	Fraction of PYR production (V4/V3)	Fraction of lactate production ( V6/V3)	Fraction of acetate production (V7 + V10)/V3	Fraction of succinate production (V16/V3)
BW25113(DE3)	3.08 ± 0.02 %	96.92 ± 0.02 %	23.46 ± 0.71 %	77.25 ± 2.66 %	2.61 ± 0.02 %
BKS8	8.53 ± 0.03 %	91.47 ± 0.02 %	19.43 ± 0.48 %	73.70 ± 1.56 %	8.29 ± 0.01 %
BKS9	12.39 ± 0.45 %	86.93 ± 1.43 %	19.95 ± 0.14 %	66.74 ± 0.08 %	12.61 ± 0.47 %
BKS10	13.66 ± 0.22 %	86.34 ± 1.10 %	18.28 ± 0.44 %	67.84 ± 2.89 %	13.66 ± 0.22 %
BKS11	45.94 ± 0.73 %	53.87 ± 0.72 %	16.97 ± 0.59 %	36.90 ± 0.32 %	55.54 ± 0.98 %
BKS15	52.31 ± 0.83 %	47.69 ± 0.67 %	14.88 ± 0.76 %	29.91 ± 0.70 %	67.20 ± 0.78 %

In strain BKS11, 45.94 % of PEP was converted to OAA (V5/V3), 2.4-fold higher than that of strain BKS10 (*p* < 0.01). As a result, the fraction of the metabolic flux to succinate (V16/V3) increased from 13.66 % in strain BKS10 to 55.54 % in strain BKS11 (Table 1) (*p* < 0.01). Meanwhile, strain BKS11 showed lower acetic fluxes ((V7 + V10)/V3). This indicated that co-overexpression of *pck*-*ecaA* could significantly enhanced the metabolic flux of PEP to OAA, and simultaneously inhibit other metabolic branches. Compared to strain BKS11, the fractions of the metabolic flux to lactate (V6/V3) and acetate ((V7 + V10)/V3) of strain BKS15 decreased by 12.32 % (*p* < 0.05) and 18.94 % (*p* < 0.01), respectively (Table 1), indicating that expression of anti-*pykF* attenuated the accumulation of lactate and acetate. At last, with a series of metabolic modifications, compared to strain BW25113(DE3), the final fraction of the metabolic flux to succinate in BKS15 was increased by 24.8 fold (*p* < 0.01) and those to lactate and acetate were decreased by 36.57 % (*p* < 0.01) and 61.28 % (*p* < 0.01), respectively.

### Anaerobic batch fermentation for succinate production

**Fig. 6:**
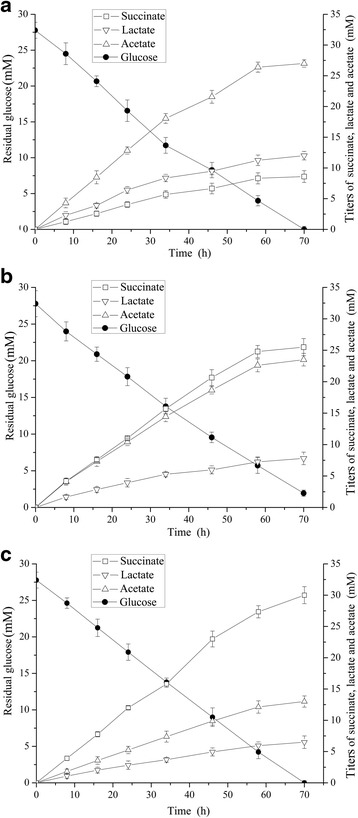
Anaerobic fermentation of engineered succinate-producing strains. **a** BKS10, **b** BKS11, **c** BKS15

To estimate the fermentation behaviors of engineered succinate-producing strains, anaerobic batch experiments were conducted. The titers, yields , specific productivities and productivities of succinate, lactate and acetate in 70 h fermentation were summarized in Table 2. As shown in Fig. 6, the distribution pattern of glucose metabolism and the production of succinate, lactate and acetate were remarkably changed. Strain BKS10 exhausted almost glucose, and accumulated large amounts of lactate and acetate, and a small amount of succinate in 70 h fermentation. Compared to strian BKS10, co-overexpression of *pck*-*ecaA* in strain BKS11 retarded glucose consumption, but achieved higher succinate production (25.51 mM), higher succinate yield (0.92 mol/mol glucose) and higher succinate specific productivity (3.96 mmol gDCW^−1^ h^−1^), increased by 1.9- (*p* < 0.01), 1.9- (*p* < 0.01) and 2.6-fold (*p* < 0.01), respectively. Moreover, the accumulation of lactate and acetate was significantly attenuated. When anti-*pykF* was further expressed in strain BKS15, glucose was completely consumed and largely distributed to succinate. Production of succinate in strain BKS15 was increased at a linear manner during the fermentation, and the specific productivity of succinate increased by 48.74 % (*p* < 0.01); the accumulation of acetate was greatly decreased, and the specific productivity of acetate decreased by 31.64 % (*p* < 0.01). Engineered strain BKS15 showed the optimal fermentation performance of higher productivity, titer and yield of succinate with the lower accumulation of lactate and acetate.

**Table 2:** Parameters of succinate production by engineered *E. coli* strians during anaerobic fermentation

Strains	Growth rate (h^−1^)	Titer (mM)			Yield (mol/mol of glucose)			Specific productivity (mmol gDCW^−1^ h^−1^)			Productivity (mmol L^−1^ h^−1^)		
		Succinate	Lactate	Acetate	Succinate	Lactate	Acetate	Succinate	Lactate	Acetate	Succinate	Lactate	Acetate
BKS10	0.071 ± 0.002	8.65 ± 0.73	12.06 ± 0.70	27.13 ± 2.56	0.31 ± 0.02	0.43 ± 0.02	0.98 ± 0.09	1.09 ± 0.06	1.47 ± 0.20	3.31 ± 0.13	0.12 ± 0.01	0.17 ± 0.01	0.39 ± 0.04
BKS11	0.052 ± 0.003^**^	25.51 ± 1.79^**^	7.82 ± 0.63^**^	23.52 ± 1.53	0.92 ± 0.06^**^	0.28 ± 0.02^**^	0.85 ± 0.05	3.96 ± 0.13^**^	1.18 ± 0.04^*^	3.54 ± 0.08	0.36 ± 0.03^**^	0.11 ± 0.01	0.34 ± 0.02
BKS15	0.043 ± 0.002^*^	30.12 ± 3.31	6.55 ± 0.33^*^	13.22 ± 1.64^**^	1.08 ± 0.11	0.24 ± 0.01^*^	0.48 ± 0.06^**^	5.89 ± 0.41^**^	1.20 ± 0.07	2.42 ± 0.19^**^	0.43 ± 0.05	0.09 ± 0.01	0.19 ± 0.02^**^

The data are shown as mean values ± standard deviation (SD) of three replicates. Asterisks indicate *p*-values (**p < 0.01, *p < 0.05) in which BKS11 was compared to BKS10 and BKS15 was compared to BKS11

## Conclusion

In this paper, PEP was selected as optimized target for increased succinate production and attenuated accumulation of byproducts in engineered *E. coli* under anaerobic conditions. By deleting *ptsG*, *pykA*, *ppc* and *maeAB* genes, we have designed and constructed initial succinate-producing *E. coli* strain. The succinate metabolic pathway was then enhanced with deletion of *sdh* and co-overexpression of *pck*-*ecaA,* resulting in succinate production of 25.51 mM. By introducing artificial sRNA of anti-*pykF*, the titer of succinate in the final optimized strain BKS15 was 30.12 mM with remarkable decrease in lactate and acetate. Metabolic flux analysis and fermentation kinetics showed that our optimization strategy could efficiently enhance the central carbon flux to succinate and decrease to byproducts. Recently, the progress in metabolic engineering suggested that limitation of cellular ATP supply and redox unbalance can be alleviated for improving succinate production in *E. coli* [41]. Combination of our strategies with those targets would further develop high succinate-producing microorganisms.

## Methods

### Bacterial strains and plasmids

*E. coli* DH5α was used for plasmids cloning and BW25113 was used as the wildtype strain for the construction of all engineered strains described in this study and succinate production. The *ecaA* gene was kindly donated by professor Jian-Min Xing, Chinese Academy of Sciences. Bacterial strains and plasmids used in this study were listed in Table 3.

**Table 3:** *E. coli* strains and plasmids used in this study

Name	Characteristics	Source
Strains		
BW25113	*lacI* ^q^ *rrnB*_T14_Δ*lac*Z_WJ16_ *hsdR514*Δ*araBAD*_AH33_	NBRP-*E. coli* at NIG
BW25113(DE3)	*lacI* ^q^ *rrnB*_T14_Δ*lac*Z_WJ16_ *hsdR514*Δ*araBAD*_AH33_dcm (DE3)	This study
BKS1	BW25113(DE3) harboring pCDF-pck	This study
BKS2	BW25113 harboring pCDF-pck	This study
BKS3	BL21(DE3) harboring pCDF-pck	This study
BKS4	BW25113(DE3) Δ*ptsG*::FRT	This study
BKS5	BKS4 Δ*pykA*::FRT	This study
BKS6	BKS5 Δ*ppc*::FRT	This study
BKS7	BKS6 Δ*maeA*::FRT	This study
BKS8	BKS7 Δ*maeB*::FRT	This study
BKS9	BKS8 Δ*sdh*::FRT	This study
BKS10	BKS9 Δ*iclR*::FRT	This study
BKS11	BKS10 harboring pCDF-pck-ecaA	This study
BKS12	BKS8 harboring pCDF-pck	This study
BKS13	BKS8 harboring pCDF-pck and pRSF-anti-pykF	This study
BKS14	BKS8 harboring pCDF-pck and pBldg-anti-pykF	This study
BKS15	BKS11 harboring pBldg-anti-pykF	This study
Plasmids		
pKD3	FRT(FLP recognition target) sites; Cm^R^	(Datsenko and Wanner 2000)
pKD46	Red recombinase expression vector; Amp^R^	(Datsenko and Wanner 2000)
pCP20	FLP expression vector; Amp^R^,Cm^R^	(Datsenko and Wanner 2000)
pETDuet-1	pBR322 ori with P_T7_; Amp^R^	Novagen
pCDFDuet-1	CDF ori with P_T7_; Str^R^	Novagen
pRSFDuet-1	RSF ori with P_T7_; Kan^R^	Novagen
pBldgbrick2	p15A ori with P_lacUV5_; Cm^R^	(Yao et al, 2013)
pCDF-pck	pCDFDute-1 with *pck*	This study
pCDF-pck-ecaA	pCDFDuet-1 with *pck* and *ecaA*	This study
pRSFM1	pRSF without RBS sequence	This study
pRSF-anti-pykF	pRSFM1 with anti*-pykF*	This study
pBldg-anti-pykF	pBldgbrick2 with anti*-pykF*	This study

### Construction of engineered strains and plasmids

Restriction endonucleases and T4 DNA ligase were purchased from Thermo Scientific (USA), High-Fidelity DNA polymerase used for PCR amplification was purchased from Transgene Biotech (Beijing, China). Appropriate restriction sites were added to 5′and 3′ ends of the primers and all primers used in this study were listed in Additional file 1: Table S4. All plasmids was constructed through the enzymatic digestion of PCR products and plasmids with appropriate restriction sites, followed by the ligation of the appropriate fragments. Clones bearing inserted gene were screened by PCR and recombinant plasmids were confirmed by DNA sequencing.

By using the lambda Red recombinase system [42], the gene coding for T7 RNA polymerase was inserted into the genome of *E. coli* BW25113. The DNA fragment containing 500 bp upstream of the *ybhB* gene, T7 RNA polymerase gene, chloramphenicol resistance cassette and 500 bp downstream of the *ybhC* gene was constructed. The detailed procedure was shown in Additional file 1: Figure S1 and the primers used were shown in Additional file 1: Table S4. This DNA fragment was then electrotransformed into *E. coli* BW25113 which expressed lambda Red system for homologous recombination. The positive clones were confirmed with primers F-ybhB and R-ybhC. Next, the chloramphenicol resistance cassette was removed with the help of pCP20 and its removal was confirmed with primers F-ybhB and R-ybhC. The function of T7 RNA polymerase in BW25113 (DE3) was verified by SDS-PAGE of BW25113 (DE3) carrying pCDF-pck, using BL21 (DE3) harboring pCDF-pck and BW25113 harboring pCDF-pck as positive and negative controls, respectively (Additional file 1: Figure S2).

All in-frame gene deletion strains were constructed in *E. coli* BW25113 (DE3) according to the procedure described previously [42] and confirmed by PCR. Briefly, for deleting *ptsG* as example, the DNA fragment containing the chloramphenicol resistance cassette for homologous recombination was amplified by PCR using F-ptsG-Q and R-ptsG-Q as primers and the plasmid pKD3 as the template. The DNA fragment was then electrotransformed into *E. coli* BW25113 (DE3) which expressed lambda Red system for homologous recombination. The replacement of *ptsG* gene was confirmed by PCR using the primers F-ptsG and R-ptsG and the removal of chloramphenicol resistance was confirmed with primers F-ptsG and R-ptsG listed in Additional file 1: Table S4. The same procedure was performed for deletions of *pykA*, *ppc*, *maeA*, *maeB, sdh*, and *iclR*.

For construction of pRSF-anti-pykF and pBldg-anti-pykF, the complementary sequence that spans to + 24 nucleotides of *pykF* coding mRNA was used as the binding sequence and was designed in the primer. In order to construct pRSF-anti-pykF, the sequence between RBS and terminator was removed from pRSFDuet-1 using primers F-RSF and R-RSF, followed by the ligation, resulting in pRSFM1. The scaffold *micC* with 24 bp binging sequence at the 5′ end [40] was amplified with primers F-RSF-anti-pykF and R-RSF-anti-pykF and cloned into the *Spe*I site of pRSFM1 (high-copy-number plasmid), and resulting in plasmid pRSF-anti-pykF. The correct construct pRSF-anti-pykF was screened by PCR using primers ACYCDuetUP1 and R-RSF-anti-pykF, and confirmed DNA sequencing. The DNA fragment containing 24 bp binding sequence and *micC* was amplified by PCR with primers F-Bldg-anti-pykF and R-Bldg-anti-pykF listed in Additional file 1: Table S4 using *E. coli* BW25113 genome as template. Then, PCR product was cloned into vector pBldgbrick2 (low-copy-number plasmid) [43] between *Hin*dIII and *Nco*I, resulting plasmid pBldg-anti-pykF. The plasmids with anti-*pykF* sequence were used to silence the expression of *pykF* gene.

### Fermentation conditions

Dual phase fermentation mode was employed [38]. For all engineered *E. coli* strains, a seed inoculum of 500 μL from an overnight 3 mL of LB culture was first inoculated at 37 °C in 250 mL shake flask containing 100 mL of liquid LB medium for aerobic growth. When the optical density (OD) reached 1.0, cells were induced with a final concentration of 0.1 mM isopropyl-β-D-thiogalactopyranoside (IPTG) and grown for another 3 h for recombinant protein expression. Then, bacterial cells were collected by centrifugation and resuspended in 150 mL shake flask containing 100 mL of fresh YM9 medium (1*M9 salts, 1 g/L yeast extract) at an initial OD of 1.0 for anaerobic fermentation. At that point, 5 g/L CaCO_3_, 2 g/L NaHCO_3_, 0.1 mM IPTG were added. Flasks were sealed with non-ventilated plugs. The cells were incubated at 37 °C on a shaker (150 rpm) and sample were collected at 40 h for analysis. For kinetic study, samples were collected at 0, 8, 16, 24, 34, 46, 58 and 70 h. Appropriate amounts of antibiotics (50 mg/L ampicillin, 30 mg/L streptomycin, 30 mg/L kanamycin) were added to media when needed.

### Analytical techniques

Cell growth was monitored by measuring the optical density (OD) at 600 nm (UV-vis spectrophotometer) and was transformed into dry cell weight using the coefficient as: dry cell mass (g L^−1^) = 0.48*OD_600_ [44]. The concentration of glucose was measured using SBA-90B biosensor (Biology Institute of ShanDong Academy of Science, China). The sample was centrifuged and the supernatant of fermentation sample was filtered through 0.2 μm syringe filter and metabolites were analyzed using an Waters 1515 differential HPLC system equipped with a Bio-Red HPX-87H HPLC column. 10 μL of sample was injected into the HPLC at column temperature of 65 °C and ran isocratically with 5 mM H_2_SO_4_ as mobile phase sat on a flow rate of 0.6 ml/min.

### Metabolic flux analysis

The metabolic network was constructed based on engineered pathways in anaerobically grown *E. coli* (Fig. 1). This network included glycolysis, TCA cycle and glyoxylate bypass (Fig. 5). As an attempt to analyze the distribution of carbon source, the fluxes through each pathway in the metabolic network were designated by V1-V16. The simplified central metabolic reactions were described in detail in Additional file 1: Table S1. According to the law of mass conversation and the quasi-steady-state assumption, these metabolic flux relationships were constructed to simplify the computational process, and shown in Additional file 1: Table S2, in which V1, V6, V16, and V7 + V10 were measurable quantities while the others were the metabolic fluxes of the corresponding intermediates. In this study, Lingo software [45] was used to obtain the solutions to distribution of metabolic fluxes that were limited by the formulas in Additional file 1: Table S2.

### Statistical analysis

The data are shown as mean values ± standard deviation (SD) of three replicates. The Student’s *t* test was used for all statistical analysis using SPSS 17.0. The *p*-value of < 0.05 and < 0.01 was considered statistically significant, more significant, respectively.

## Abbreviations

ATP, adenosine triphosphate; G3P, Glyceraldehyde 3-P; Glf, glucose facilitator; IPTG, isopropyl-β-D-thiogalactopyranoside; NADH, Nicotinamide adenine dinucleotide; OAA, oxaloacetate; PCK, PEP carboxykinase; PEP, phosphoenolpyruvate; PPC, PEP carboxylase; PTS, PEP carbohydrate phosphotransferase system; PYC, pyruvate carboxylase; PYR, pyruvate; SDH, succinate dehydrogenase

## Additional file

Additional file 1: Figure S1. Assembly of DNA fragment for inserting the T7 RNA polymerase into BW25113 genome. **Figure S2.** SDS-PAGE of protein expression of strain BKS1, BKS2 and BKS3. **Table S1.** The simplified central metabolic reaction of engineered *E. coli* anaerobically grown in glucose. **Table S2.** Stoichiometric relationships for fluxes of metabolic reactions in anaerobic growth of *E. coli*. **Table S3.** Metabolic fluxes (mM gDCW^−1^ h^−1^) of engineered strains based on the anaerobic fermentation results at 40 h. **Table S4.** Primers used in this study. (DOCX 128 kb)
