# Supplementary material for: Targeted optimization of central carbon metabolism for engineering succinate production in Escherichia coli
Source: BMC Biotechnol. 2016 Jun 24;16:52. doi: 10.1186/s12896-016-0284-7 (PMC4919853; doi:10.1186/s12896-016-0284-7)
Supplement: Additional file 1: Figure S1. — Assembly of DNA fragment for inserting the T7 RNA polymerase into BW25113 genome. Figure S2. SDS-PAGE of protein expression of strain BKS1, BKS2 and BKS3. Table S1. The simplified central metabolic reaction of engineered E. coli anaerobically grown in glucose. Table S2. Stoichiometric relationships for fluxes of metabolic reactions in anaerobic growth of E. coli. Table S3. Metabolic fluxes (mM gDCW−1 h−1) of engineered strains based on the anaerobic fermentation results at 40 h. Table S4. Primers used in this study. (DOCX 128 kb) [file 12896_2016_284_MOESM1_ESM.docx]

Additional file 1

Targeted optimization of central carbon metabolism for engineering succinate production in *Escherichia coli*

Ying Zhao^a,b,c^, Chang-Song Wang^a,b,c^, Fei-Fei Li^a,b,c^, Zhen-Ning Liu^a, b, c^, Guang-Rong Zhao^a.b.c,^*

^a^Department of Pharmaceutical Engineering, School of Chemical Engineering and Technology, Tianjin University, Tianjin 300072, China;

^b^Key Laboratory of Systems Bioengineering, Ministry of Education Tianjin 300072, China;

^c^SynBio Research Platform, Collaborative Innovation Center of Chemical Science and Engineering , Tianjin 300072, China.

The document contains:

Supplementary Figure S1………………………………………………………………2

Supplementary Figure S2………………………………………………………………4

Supplementary Table S1 ………………………………………………………………5

Supplementary Table S2 ………………………………………………………………6

Supplementary Table S3 ………………………………………………………………7

Supplementary Table S4 ………………………………………………………………8

Abbreviations ………………………………………………………………14

**Supplementary figures**


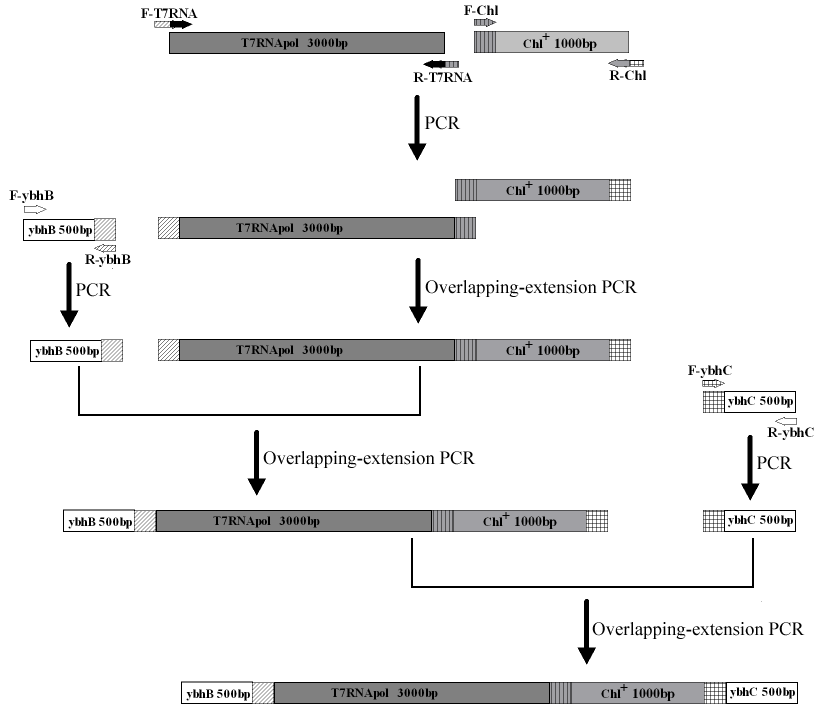


**Figure S1.** Assembly of DNA fragment for inserting the T7 RNA polymerase into BW25113 genome.

T7 RNA polymerase gene with *lacUV5* promoter was amplified from *E. coli* BL21(DE3) with primers F-T7RNA and R-T7RNA. Chloramphenicol resistance cassette was amplified from plasmid pKD3 with primers F-Chl and R-Chl. 500 bp upstream of the *ybhB* gene and 500 bp downstream of the *ybhC* gene as homologous regions were amplified from genome of *E. co*li BW25113 using primers F-ybhB, R-ybhB and F-ybhC, R-ybhC, respectively. All the annealing temperature for PCR procedures of these four DNA fragment were 56℃. After obtaining the four individual DNA fragment, T7 RNA polymerase fragment and chloramphenicol resistance cassette were first assembled into one fragment by overlapping extension PCR using primers F-T7RNA and R-Chl, resulting in DNA fragment T7RNA-Chl. Using primers F-ybhB and R-Chl, DNA fragment 500 bp upstream of the *ybhB* gene and T7RNA-Chl were extended into one DNA fragment ybhB-T7RNA-Chl, followed by overlaping extension RCR procedure with DNA fragment ybhC using primers F-ybhB and R-ybhC, resulting in the final homologous recombination DNA fragment ybhB-T7RNA-Chl-ybhC.


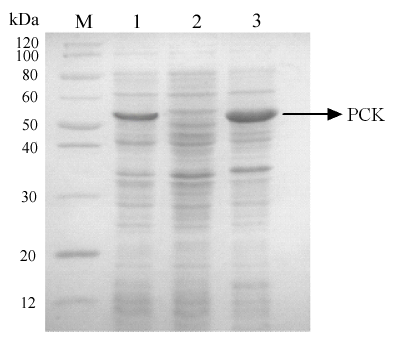


**Figure S2.** SDS-PAGE of protein expression of strain BKS1, BKS2 and BKS3.

1.total cell protein of strain BKS1, 2.totel cell protein of strain BKS2, 3.total cell protein of strain BKS3, M. marker standards. All of the three strains were induced with 1mM IPTG.

**Supplementary tables**

**Table S1** The simplified central metabolic reaction of engineered E.coli anaerobically grown in glucose

| metabolic reactions | gene names |
| --- | --- |
| 1. Glucose+PEP=glucose 6-P+pyruvate (for wildtype E. coli)  1. Glucose +ATP=glucose 6-P+ADP (for strains with deltion of *ptsG*) | *PTS* sysytem  *glk* |
| 2. Glucose 6-P+ATP=2 glyceraldehyde 3-P+ADP | *pgi, pfkA, fda, tpia* |
| 3. Glyceraldehyde 3-P+NAD^+^+Pi+ADP=PEP+NADH+H^+^+ATP+H_2_O | *gapA, pgk, pgm, eno* |
| 4. PEP+ADP=pyruvate+ATP | *pyk* |
| 5. PEP+CO_2_=OAA+P | *pck* |
| 6. Pyruvate+NADH=lactate+NAD^+^ | *ldhA* |
| 7. Pyruvate+ubiquinone+H_2_O=acetate+CO_2_+ubiquinol | *poxB* |
| 8.Pyruvate+NAD^+^=NADH+CO_2_+acetyl-CoA | *pdh* |
| 9. Malate+NAD^+^=PYR+CO_2_+NADH | *maeAB* |
| 10. Acetyl-CoA+Pi+ADP=acetate+HSCoA+ATP | *pta，ackA* |
| 11. 2 Acetyl-CoA+OAA+H_2_O=succinate+malate+CoA+HSCoA | *gltA, acnAB, aceBAK* |
| 12. Oxaloactetate+NADH=malate+NAD^+^ | *mdh* |
| 13. Malate=fumarate+H_2_O | *fumABC* |
| 14. Succinate+FAD=fumrate+FADH_2_ | *sdh* |
| 15. Fumarate+NADH+Pi+ADP=succinate+NAD^+^+ATP | *frd* |
| 16. Succinate=excreted succinate |  |

**Table S2** Stoichiometric relationships for fluxes of metabolic reactions in anaerobic growth of *E.coli*

| Metabolite | Net conversion |
| --- | --- |
| Glucose consumption | V1 |
| Excreted succinate | V16 |
| Excreted lactate | V6 |
| Excreted acetate | V7+V10 |
| G6P | V1-V2=0 |
| G3P | V3-2V2=0 |
| PEP | V3-V4-V5=0 |
| PYR | V4+V9-V6-V7-V8 =0 |
| Acetyl-CoA | V8-2V11-V10=0 |
| OAA | V5-V11-V12=0 |
| Malate | V11+V12-V13-V9=0 |
| Fumarate | V13+V14-V15=0 |
| Succinate | V14+V16-V11-V15=0 |

**Table S3** Metabolic fluxes (mM gDCW^-1^ h^-1^) of engineered strains based on the anaerobic fermentation results at 40h

| Flux | To | BW25113  (DE3) | BKS8 | BKS9 | BKS10 | BKS11 | BKS15 |
| --- | --- | --- | --- | --- | --- | --- | --- |
| V1 | Glucose uptake | 2.11±0.15 | 2.11±0.18 | 2.18±0.16 | 2.27±0.11 | 2.71±0.24 | 3.46±0.31 |
| V2 | G3P | 2.11±0.15 | 2.11±0.18 | 2.18±0.16 | 2.27±0.11 | 2.71±0.24 | 3.46±0.31 |
| V3 | PEP | 4.22±0.30 | 4.22±0.36 | 4.36±0.32 | 4.54±0.22 | 5.42±0.48 | 6.92±0.62 |
| V4 | PYR | 4.09±0.29 | 3.86±0.33 | 3.79±0.34 | 3.92±0.24 | 2.92±0.22 | 3.3±0.25 |
| V5 | OAA | 0.13±0.01 | 0.36±0.03 | 0.54±0.02 | 0.62±0.02 | 2.49±0.26 | 3.62±0.37 |
| V6 | Lactate | 0.99±0.10 | 0.82±0.05 | 0.87±0.07 | 0.83±0.06 | 0.92±0.05 | 1.03±0.04 |
| V7 | Acetate | 1.63±0.06 | 1.49±0.10 | 1.26±0.06 | 1.54±0.14 | 1.00±0.08 | 0 |
| V8 | Acetyl-CoA | 1.63±0.06 | 1.62±0.10 | 1.65±0.15 | 1.54±0.14 | 1.00±0.08 | 2.27±0.21 |
| V9 | PYR | 0.02±0.002 | 0 | 0 | 0 | 0 | 0 |
| V10 | Acetate | 1.63±0.06 | 1.62±0.10 | 1.65±0.15 | 1.54±0.14 | 1.00±0.08 | 2.07±0.19 |
| V11 | Glyoxylase succinate | 0 | 0 | 0 | 0 | 0 | 0.1±0.007 |
| V12 | Malate | 0.13±0.01 | 0.35±0.03 | 0.55±0.02 | 0.62±0.02 | 2.49±0.26 | 3.52±0.36 |
| V13 | Fumatate | 0.11±0.007 | 0.35±0.03 | 0.55±0.02 | 0.62±0.02 | 2.49±0.26 | 3.62±0.37 |
| V14 | Fumatate | 0.04±0.002 | 0.06±0.004 | 0 | 0 | 0 | 0 |
| V15 | Intracellular succinate | 0.15±0.005 | 0.41±0.03 | 0.55±0.02 | 0.62±0.02 | 3.01±0.31 | 4.64±0.47 |
| V16 | Excreted succinate | 0.11±0.007 | 0.35±0.03 | 0.55±0.02 | 0.62±0.02 | 3.01±0.31 | 4.65±0.47 |

**Table S4** Primers used in this study

| Name | Sequence 5’-3’ | Use |
| --- | --- | --- |
| F-T7RNA | TTTACCTTCCCGTTTCGCTCGTATCAAGGTATTTTATGCG | cloning T7 RNA polymerase |
| R-T7RNA | GAAGCAGCTCCAGCCTACTCACTCATTAGGCACCCC |  |
| F-Chl | GTAGGCTGGAGCTGCTTC | cloning chloramphenicol resistance cassette |
| R-Chl | CTGCTTTTTTATACTAACTTGTAGGCTGGAGCTGCTTC |  |
| F-ybhB | AGAAAGGAGGGTTCATGAAA | 500 bp upstream of the *ybhB* gene |
| R-ybhB | GAGCGAAACGGGAAGGTAAA |  |
| F-ybhC | AAGTTAGTATAAAAAAGCAG | 500 bp downstream of the *ybhC* gene |
| R-ybhC | ATCAAGGGAAAGCCCAATCT |  |
|  |  |  |
|  |  |  |
| F-ptsG-Q | CCATACTCAGGAGCACTCTCAATTATGTTTAAGAATGCATTTGCTAACCTGTGTAGGCTGGAGCTGCTTC | cloning DNA fragment for insertional inactivation of *ptsG* gene |
| R-ptsG-Q | GCCTTAGTCTCCCCAACGTCTTACGGATTAGTGGTTACGGATGTACTCATCATGGGAATTAGCCATGGTCC |  |
| F-ptsG | ATCGGTTACTGGTGGAAACTG | confirming the insertional inactivation of *pts*G gene and removal of chloramphenicol resistance |
| R-ptsG | GTGGATGGGACAGTCAGTAAAG |  |
| F-pykA-Q | TCAGTCAACGGAGTATTACATGTCCAGAAGGCTTCGCAGAACAAAAATCGGTGTAGGCTGGAGCTGCTTC | cloning DNA fragment for insertional inactivation of *pykA* gene |
| R-pykA-Q | TTACTCTACCGTTAAAATACGCGTGGTATTAGTAGAACCCACGGTACTCATGGGAATTAGCCATGGTCC |  |
| F-pykA | TGGTGGTGCTGATTTCTCAC | confirming the insertional inactivation of *pykA* gene and removal of chloramphenicol resistance |
| R-pykA | ACCGCTGTTTCCGATTTATG |  |
|  |  |  |
| F-ppc-Q | GATAAGATGGGGTGTCTGGGGTAATATGAACGAACAATATTCCGCATTGCGTGTAGGCTGGAGCTGCTTC | cloning DNA fragment for insertional inactivation of *ppc* gene |
| R-ppc-Q | CACGAGGGTTTGCAGAAGAGGAAGATTAGCCGGTATTACGCATACCTGCATGGGAATTAGCCATGGTCC |  |
| F-ppc | CGACCGACAGTGACTCAAA | confirming the insertional inactivation of *ppc* gene and removal of chloramphenicol resistance |
| R-ppc | ATAGCACCACGCCGATTA |  |
| F-maeA-Q | GATATTCAAAAAAGAGTGAGTGACATGGAACCAAAAACAAAAAAACAGCGGTGTAGGCTGGAGCTGCTTC | cloning DNA fragment for insertional inactivation of *maeA* gene |
| R- maeA-Q | GCCTTCACTACCGGGCGCAGGC TTAGATGGAGGTACGGCGGTAGTCGCGGCATGGGAATTAGCCATGGTC |  |
| F- maeA | CAGACACGGAGAATCACTAT | confirming the insertional inactivation of *maeA* gene and removal of chloramphenicol resistance |
| R- maeA | AGACCATCTGGCACTTTTAC |  |
| F-maeB-Q | GTTACGTGAAAGGAACAACCAAATGGATGACCAGTTAAAACAAAGTGCACGTGTAGGCTGGAGCTGCTTC | cloning DNA fragment for insertional inactivation of *maeB* gene |
| R- maeB-Q | GGTAAGCGTGAGAGTTAAAAAAAATTACAGCGGTTGGGTTTGCGCTTCTACATGGGAATTAGCCATGGTCC |  |
| F- maeB | CTCGTTCATAGGAAATACTC | confirming the insertional inactivation of *maeB* gene and removal of chloramphenicol resistance |
| R- maeB | GGTTTGTGATAACGCTTCTT |  |
| F-sdh-Q | TGTGTGATGAAATTGCCAGTCAGAGAATTTGATGCAGTTGTGATTGGTGCGTGTAGGCTGGAGCTGCTTC | cloning DNA fragment for insertional inactivation of *sdh* gene |
| R-sdh-Q | GCTTGCGCGTCTTATCAGGCCTACGGTTTACGCATTACGTTGCAACAACACATGGGAATTAGCCATGGTC |  |
| F-sdh | GCAGGAGTCCTCGTATGGTA | confirming the insertional inactivation of *sdh* gene and removal of chloramphenicol resistance |
| R-sdh | CGCCAGAGAGGTAAGAAGAG |  |
|  |  |  |
| F-iclR-Q | GATACAGAAAAAAGAGACTGTC  ATGGTCGCACCCATTCCCGCGAAACGCGGTAGGCTGGAGCTGCTTC | cloning DNA fragment for insertional inactivation of *iclR* gene |
| R-iclR-Q | TATTGCCTCTGCCCGCCAGAAAAAGTCAGCGCATTCCACCGTACGCCAGCCATGGGAATTAGCCATGGTC |  |
| F-iclR | CCGACAGGGATTCCATCTGG | confirming the insertional inactivation of *iclR* gene and removal of chloramphenicol resistance |
| R-iclR | TATGACGACCATTTTGTCTA |  |
| F-pck | CGCGGATCCGATGCGCGTTAACAATG | constructing pCDF-pck-ecaA |
| R-pck | CGCGGATCCATCATTACAGTTTCGGA |  |
| F-ecaA | CCAATTCCATATGAGTAGTAC |  |
| R-ecaA | CGGGGTACCTTAAATGGCTTC |  |
| F-pRSF | CTAGACTAGTCTGGTAAAGAAACC | constructing pRSFM1 |
| R-pRSF | CTAGACTAGAACAAAATTATTTCTACAGGGG |  |
| F-RSF-anti-pykF | CTAGACTAGTGCAAACAATTTTGGTCTTTTTCATTTTCTGTTGGGCCATTGCATTGC | constructing pRSF-anti-pykF |
| R-RSF-anti-pykF | CTAGACTAGTAAAAAAAGCCCGGACGACTGTTC |  |
| ACYCDuetUp1 | GGATCTCGACGCTCTCCCT | confirming the correct ligationof pRSF-anti-pykF |
| F-Bldg-anti-pykF | CCCAAGCTTGCAAACAATTTTGGTCTTTTTCATTTTCTGTTGGGCCATTGCATTGC | constructing pBldg-anti-pykF |
| R-Bldg-anti-pykF | CATGCCATGGAAAAAAAGCCCGGACGACTGTTC |  |

**Abbreviations**

Genes coding the corresponding enzymes: *ybhB*, putative kinase inhibitor protein; *ybhC*, acyl-CoA thioesterase; *ptsG*, glucose phosphotransferase; *pykA*, pyruvate kinase II; *ppc*, PEP carboxylase; *maeAB*, malic enzyme; *sdh*, succinate dehydrogenase; *iclR*, transcriptional repressor IclR; *pck*, PEP carboxykinase; *ecaA*, carbonic anhydrase; *pykF*, pyruvate kinase I; *glk*, glucokinase; *pgi*, glucosephosphate isomerase; *pfkA*, 6-phosphofructokinase I; *fda*, fructose-bisphosphate aldolase; *tpia*, triosephosphate isomerase; *gapA*, glyceraldehyde-3-phosphate dehydrogenase A; *pgk*, phosphoglycerate kinase; *pgm*, phosphoglucomutase; *eno*, enolase; *ldhA*, lactate dehydrogenase; *poxB*, pyruvate oxidase; *pdh*, pyruvate dehydrogenase; *pta*, phosphotransacetylase; *ackA*, acetate kinase; *gltA*, glutamate; *acnAB*, aconitate hydratase; *aceA*, isocitrate lyase; *aceB*, malate synthase; *aceK*, isocitrate dehydrogenase kinase/phosphatase; *mdh*, malate dehydrogenase; *fumABC*, fumaraseABC; *frd*, fumarate reductase.
